# Alzheimer Disease Pathology and Neurodegeneration in Midlife Obesity: A Pilot Study

**DOI:** 10.14336/AD.2023.0707

**Published:** 2024-08-01

**Authors:** Mahsa Dolatshahi, Paul K Commean, Farzaneh Rahmani, Jingxia Liu, LaKisha Lloyd, Caitlyn Nguyen, Nancy Hantler, Maria Ly, Gary Yu, Joseph E Ippolito, Claude Sirlin, John C Morris, Tammie L.S Benzinger, Cyrus A Raji

**Affiliations:** ^1^Mallinckrodt Institute of Radiology, Washington University in St. Louis, St. Louis, Missouri, USA.; ^2^Washington University School of Medicine, Division of Public Health Sciences, Department of Surgery, St. Louis, Missouri, USA.; ^3^Department of Biochemistry and Molecular Biophysics, Washington University School of Medicine, St. Louis, Missouri, USA.; ^4^Liver Imaging Group, Department of Radiology, University of California, San Diego, La Jolla, California, USA.; ^5^Department of Neurology, Washington University School of Medicine, St Louis, Missouri, USA.; ^6^Knight Alzheimer Disease Research Center, Washington University School of Medicine, Saint Louis, Missouri, USA.; ^7^Department of Neurosurgery, Washington University School of Medicine, St Louis, Missouri, USA.

**Keywords:** Alzheimer disease, obesity, visceral adipose tissue, beta-amyloid, PET, MRI

## Abstract

Obesity and excess adiposity at midlife are risk factors for Alzheimer disease (AD). Visceral fat is known to be associated with insulin resistance and a pro-inflammatory state, the two mechanisms involved in AD pathology. We assessed the association of obesity, MRI-determined abdominal adipose tissue volumes, and insulin resistance with PET-determined amyloid and tau uptake in default mode network areas, and MRI-determined brain volume and cortical thickness in AD cortical signature in the cognitively normal midlife population. Thirty-two middle-aged (age: 51.27±6.12 years, 15 males, body mass index (BMI): 32.28±6.39 kg/m2) cognitively normal participants, underwent bloodwork, brain and abdominal MRI, and amyloid and tau PET scan. Visceral and subcutaneous adipose tissue (VAT, SAT) were semi-automatically segmented using VOXel Analysis Suite (Voxa). FreeSurfer was used to automatically segment brain regions using a probabilistic atlas. PET scans were acquired using [11C]PiB and AV-1451 tracers and were analyzed using PET unified pipeline. The association of brain volumes, cortical thicknesses, and PiB and AV-1451 standardized uptake value ratios (SUVRs) with BMI, VAT/SAT ratio, and insulin resistance were assessed using Spearman’s partial correlation. VAT/SAT ratio was associated significantly with PiB SUVRs in the right precuneus cortex (p=0.034) overall, controlling for sex. This association was significant only in males (p=0.044), not females (p=0.166). Higher VAT/SAT ratio and PiB SUVRs in the right precuneus cortex were associated with lower cortical thickness in AD-signature areas predominantly including bilateral temporal cortices, parahippocampal, medial orbitofrontal, and cingulate cortices, with age and sex as covariates. Also, higher BMI and insulin resistance were associated with lower cortical thickness in bilateral temporal poles. In midlife cognitively normal adults, we demonstrated higher amyloid pathology in the right precuneus cortex in individuals with a higher VAT/SAT ratio, a marker of visceral obesity, along with a lower cortical thickness in AD-signature areas associated with higher visceral obesity, insulin resistance, and amyloid pathology.

## INTRODUCTION

Obesity is a risk factor for multiple medical conditions and a key driver of morbidity and mortality globally [1]. Obesity in midlife, defined as age 40 to 60 years and body mass index (BMI) ≥30.0 kg/m2, is a risk factor for Alzheimer disease [2]. It is speculated that the chronic low-grade pro-inflammatory state induced by midlife obesity might promote AD pathology and increases the risk for clinical dementia [3]. Nevertheless, the underlying processes and mechanisms responsible for the link between obesity and AD are not clear.

A series of studies have assessed the association between obesity and brain structure with respect to AD-related alterations. A study of more than twelve thousand midlife and older individuals from the UK Biobank data showed increased body fat mass was associated with reduced subcortical gray matter (GM) volumes in males [4]. Also, it was demonstrated that higher baseline BMI was associated with lower frontal GM volume at baseline as well as longitudinal atrophy in temporal and occipital GM in older adults [5]. Another study showed similar patterns of brain atrophy and AD pathology in obesity compared to AD patients matched by age and sex [6]. Interestingly, although obesity in midlife is associated with lower brain volumes and a higher risk of AD, obesity in late life (after age 70 years) is associated with decreased AD risk [2, 7] and higher BMI is negatively associated with cortical β-amyloid content in older adults [8-10]. In addition, higher body fat content, lower muscle mass, and higher fat-to-muscle ratio were associated with better cognitive performance and lower risk of all-cause dementia in elderly participants, especially in males [11, 12].

From an adipose tissue distribution perspective, central obesity as measured through increased sagittal abdominal diameter, waist circumference, abdominal fat, and fat-to-muscle ratio, irrespective of BMI, is associated with an increased risk of dementia decades later [13, 14]. In people with abdominal obesity, there is increased interleukin-6 and insulin along with reduced leptin levels in portal vein suggesting that in this population visceral adipose tissue (VAT) is correlated to insulin resistance and systemic inflammation, the mechanisms that are thought to contribute to AD pathology [15, 16]. Kim et al. observed that in older adults a higher visceral adipose tissue glucose metabolism, as a surrogate marker of VAT dysfunction and inflammatory status in obesity, is associated with increased cerebral amyloid-β (Aβ) burden [17]. Also, peripheral insulin-resistant glucose metabolism, which is associated with abdominal obesity [18, 19], is associated with altered brain metabolism and volume in AD-signature areas, such as the hippocampus [18, 19]. These findings suggest obesity-associated insulin resistance and inflammation in midlife contribute to AD pathology and neurodegeneration.

However, the relationship between abdominal fat components, i.e. visceral and subcutaneous adipose tissue (VAT and SAT, respectively), especially in midlife, and the development of AD pathology is not known. Visceral to subcutaneous fat ratio (VAT/SAT ratio) is commonly used as a measure of visceral obesity and an increased VAT/SAT ratio is associated with insulin resistance [20], commonly assessed by the Homeostatic Model Assessment for Insulin Resistance (HOMA-IR). It is suggested that amyloid peptides spread trans-synaptically through the interconnected areas, especially in default-mode network (DMN) regions [21]. In line with this, previous studies have shown that in cognitively normal individuals, DMN areas especially the posterior cingulate cortex, precuneus cortex, and medial orbitofrontal cortex, are affected by amyloid pathology [22, 23], which is associated with functional connectivity within DMN [22]. Similar regions show altered glucose metabolism, in association with functional connectivity [3], further emphasizing the susceptibility of DMN areas to AD-related changes.

We hypothesized that obesity (BMI), visceral adiposity (VAT/SAT ratio), and insulin resistance (HOMA-IR) in midlife (40-60 years) are associated with cortical beta-amyloid deposition, assessed by PET tracers, in the DMN areas. We also hypothesized that neurodegeneration, assessed as brain volume and cortical thickness by using MRI, in the cortical signature areas showing atrophy in AD [24], are associated with VAT/SAT ratio, HOMA-IR, and BMI even in middle-aged adults. Given the sex differences in abdominal fat distribution [25], we further investigated these associations in males and females separately. We also evaluated the associations between regional SUVRs and cortical thickness, based on the primary results.

## MATERIALS AND METHODS

### Participants

This study was done with IRB approval at our institution (IRB# 202102186) and all participants provided written informed consent before their participation. We recruited cognitively normal (Mini Mental Status Exam (MMSE) of 25 or higher, and/or Clinical Dementia Rating Scale (CDR) = 0) midlife individuals, aged 40-60 years. The Research Participant Registry/Volunteers for Health (VFH) Program at Washington University in St. Louis (https://vfh.wustl.edu) was utilized for recruitment purposes. Also, referral sources for relevant participants included the Washington University Knight Alzheimer Disease Research Center (ADRC), and the Center for Human Nutrition. The participants consented to undergo MRI and to complete molecular neuroimaging PET scans, including [11C]PiB and 18F-AV-1451 (Flortaucipir). Exclusion criteria were prior bariatric surgery, active enrollment in a bariatric surgery program, currently receiving an active obesity study drug (or placebo), current participation in an obesity clinical trial, contraindications to MRI or PET scan (e.g., claustrophobia, certain incompatible electronic medical devices that make it potentially unsafe for the individual to participate, or women who were currently pregnant or breast-feeding). Any of the volunteers fulfilling the inclusion and exclusion criteria were included. All demographic, anthropometric, clinical, laboratory, and imaging data were directly gathered by our research staff at Washington University in Saint Louis.

### Glucose Tolerance Studies

An oral glucose tolerance test was performed on all participants except those who already fulfilled the standard criteria for diabetes mellitus. These included a history of type 1 or 2 diabetes mellitus, prior documentation of fasting plasma glucose above 7.0 mmol/L, two or more occasions of clinical symptoms of diabetes mellitus e.g. polydipsia, polyuria, ketonuria, and rapid weight loss with a random plasma glucose above 11.1 mmol/L. Insulin resistance was calculated using HOMA-IR. HOMA-IR was calculated according to the formula: fasting insulin (microU/L) × fasting glucose (nmol/L)/22.5 [26]. Obesity was characterized as a BMI of 30 kg/m2 or higher.

### Abdominal MRI Acquisition & Processing

Abdominal MR images were collected using a Siemens Prisma Fit 3T scanner. A transverse T1 two-dimensional fast low-angle shot (fl2d) scan of the torso was obtained starting at the spine vertebral bone location S1-L5 and proceeding proximally for 22 slices (TR = 286 ms, TE = 3.34 ms, thickness = 10 mm). The T1 fl2D MR Dicom images were processed using an in-house developed Matlab-based program named VOXel Analysis Suite (Voxa), which performs automatic abdominal adipose tissue identification (white voxels) for eight slices (4 to 11). The torso subcutaneous and visceral adipose tissue (SAT and VAT) images were normalized to brighten the darker intensities and darken the brighter intensities using Matlab’s black and white distribution methods with a Euclidean distance transformation. The torso was identified using a radial region growing method from the centroid of the bounding box containing the torso. The non-VAT subregion within the torso was identified using dark intensities inside the SAT region and outside of the VAT region. Erosion, fill, and dilation methods were used to separate the bright VAT intensity values from the SAT bright intensity values. Manual editing of the SAT and VAT was used to remove or add any areas where the adipose was not properly automatically classified. Adipose tissue in the spinal region was removed manually (Supplemental Figure 1). Voxa automatically calculates the SAT and VAT volumes. Based on the SAT/VAT abdominal MRI volume measurements, VAT/SAT ratio was calculated as a measure of visceral obesity that is considered to be associated with cardiometabolic risk and glucose intolerance [20].

### Brain MRI Acquisition & Processing

T1-weighted brain MR images were acquired using a Siemens Prisma Fit 3T scanner (TR = 2500 ms, TE = 2.9 ms, thickness = 10 mm). The T1-weighted MRI scans were processed through the FreeSurfer image analysis suite 7.1.1 (http://surfer.nmr.mgh.harvard.edu). Free-Surfer processing included reconstruction of brain structures by segmentation of the subcortical white matter and deep gray matter volumetric structures, extraction of the cortical surfaces, and parcellation of cortical regions. The automatic segmentations were visually evaluated for quality control by a post-doctoral research fellow with one year of image processing experience. If the editing criteria were not met, manual editing was performed, and the reconstruction was rerun based on the edited images and then the brain volumes and cortical thicknesses for each region of interest were retrieved.

### Amyloid and Tau PET Image Acquisition & Processing

Dynamic amyloid imaging was performed with a bolus injection of ~15 mCi of [11C]PiB, followed by a 60-min scan. For tau PET imaging, a single intravenous bolus of between 7.2-10.8 mCi of AV-1451 was administered followed by a 105-min scan. PET imaging analyses were performed using the PET unified pipeline (PUP, https://github.com/ysu001/PUP) [27]. The standardized uptake value ratios (SUVRs) were calculated using FreeSurfer-defined ROIs with cerebellar cortex as a reference region and using a 30-60 min post-injection window and a 50-70 min post-injection window for PiB and AV-1451 respectively. To correct for signal distortions caused by the low resolution of PET images and partial volume effects, the regional spread function (RSF) technique was used [28].

### Statistical Analysis

PiB and AV-1451 SUVRs in default mode network regions (including the medial orbitofrontal cortex, posterior cingulate cortex, entorhinal cortex, precuneus cortex, and inferior parietal lobule) were considered as the outcome variables. For brain volumes and cortical thickness, a priori hypothesis was based on the regions showing atrophy or reduced thickness in late-onset or autosomal dominant AD (Supplemental Table 1). Normality assumption was assessed using the Shaprio-Wilks test and used to guide further statistical testing. When the normality assumption was met, linear regression model was used; Spearman's partial correlation was used otherwise. Associations of BMI, VAT/SAT ratio, and HOMA-IR with outcome variables were assessed using Spearman's partial correlation with age and sex as covariates, because the normality assumption was not met. Given the sex effects on obesity and abdominal fat metrics, further analyses in sex subgroups (men and women) were done. Based on the results of regional SUVRs association with abdominal obesity, further Spearman’s partial correlation analyses considering cortical thicknesses and regional SUVRs were performed. All statistical analyses were performed using R version 4.2.2 (https://cran.r-project.org/). Statistical analyses for brain volume and cortical thickness as outcome variables for each predictor variable (consisting of 28 comparisons) were corrected for multiple comparisons using the Benjamini-Hochberg procedure at the false discovery rate of 0.05 [29]. For analyses with PiB and AV-1451 SUVRs as outcome variables, no correction for multiple comparisons was performed due to the interconnected nature of default-mode network areas, not fulfilling the a priori assumption of independent observations [30].

**Table 1:** Characteristics of the sample based on each type of brain scans.

	MRI (n=32)	PiB (n=21)	AV-1451 (n=22)
**Age, years (mean; SD)**	51.27 (6.12)	51.24 (5.93)	51.50 (5.92)
**Education, years (mean; SD)**	15.97 (2.63)	15.75 (2.38)	15.86 (2.37)
**Sex, males (n, %)**	15 (46.9)	11 (52.4)	11 (50.0)
**Hypertension (n, %)**	7 (21.87)	4 (19.04)	4 (18.18)
**Diabetes Mellitus (n, %)**	2 (6.25)	1 ( 4.76)	1 (4.55)
**Race (n, %)** **White/ Caucasian** **Black/ African American**	19 (59.37) 13 (40.62)	13 (61.90) 8 (38.10)	14 (63.64) 8 (36.36)
**Waist circumference, cm (mean; SD)**	104.94 (19.10)	102.84 (21.52)	103.58 (21.16)
**Weight, kg (mean; SD)**	91.66 (18.13)	90.54 (19.84)	91.42 (19.70)
**Height, cm (mean; SD)**	169.48 (9.64)	170.36 (8.33)	169.75 (8.56)
**BMI, kg/m2 (mean; SD)**	32.28 (6.39)	31.80 (6.65)	32.32 (6.93)
**Systolic blood pressure, mmHg (mean; SD)**	121.25 (12.90)	120.61 (12.05)	121.26 (12.05)
**Diastolic blood pressure, mmHg (mean; SD)**	76.75 (8.78)	76.50 (8.79)	76.74 (8.60)
**Cholesterol, mg/dL (mean (SD))**	182.87 (36.74)	188.10 (28.26)	186.86 (28.13)
**Triglyceride, mg/dL (mean (SD))**	107.60 (62.71)	102.70 (52.85)	113.71 (72.12)
**HDL, mg/dL (mean (SD))**	53.50 (15.66)	54.35 (15.37)	53.86 (15.15)
**LDL, mg/dL (mean (SD))**	107.77 (33.93)	113.05 (23.99)	110.10 (27.02)
**Cholesterol /HDL ratio (mean (SD))**	3.73 (1.28)	133.70 (26.69)	132.95 (26.24)
**Fasting glucose, mg/dL (mean (SD))**	97.79 (22.80)	3.80 (1.20)	3.81 (1.17)
**Glucose tolerance, mg/dL (mean (SD))**	133.85 (44.66)	97.69 (27.18)	98.04 (26.41)
**HbA1C, % (mean (SD))**	5.80 (0.79)	135.27 (43.15)	138.01 (43.44)
**Fasting insulin, µU/L (mean (SD))**	15.25 (12.56)	5.86 (0.90)	5.88 (0.88)
**HOMA-IR (mean (SD))**	3.93 (3.67)	3.07 (2.84)	3.64 (3.66)
**MMSE score (mean (SD))**	29.14 (1.27)	29.58 (0.69)	29.55 (0.69)
**Subcutaneous fat, cm3 (mean (SD))**	2871.33 (1212.68)	2891.86 (1303.57)	2892.91 (1272.16)
**Visceral fat, cm3 (mean (SD))**	1137.77 (612.43)	1171.13 (630.57)	1184.96 (618.78)
**Visceral/ Subcutaneous fat ratio (mean (SD))**	0.45 (0.27)	0.46 (0.27)	0.46 (0.26)

BMI: Body Mass Index, HbA1C: Hemoglobin A1C, HDL: High-density lipoprotein, HOMA-IR: Homeostatic Model Assessment for Insulin Resistance, LDL: Low-density lipoprotein, MMSE: Mini-Mental State Exam.

## RESULTS

### Participants Characteristics

In total 36 participants were enrolled in the study and their data was utilized for analysis. After running FreeSurfer on T1-weighted brain MRIs and conducting edits, a total of 32 participants’ images (out of 36 participants) passed quality control. All of these participants had BMI and abdominal adipose tissue measurements but only 23 of them had insulin resistance measures available. Among the 32 participants passing FreeSurfer segmentation quality control, PUPs for all participants with available PET scans passed the quality control. A total of 21 PiB scans and a total of 22 AV-1451 scans were available from the 32 participants and the SUVR data for these scans were used in the analysis.

**Figure 1.**
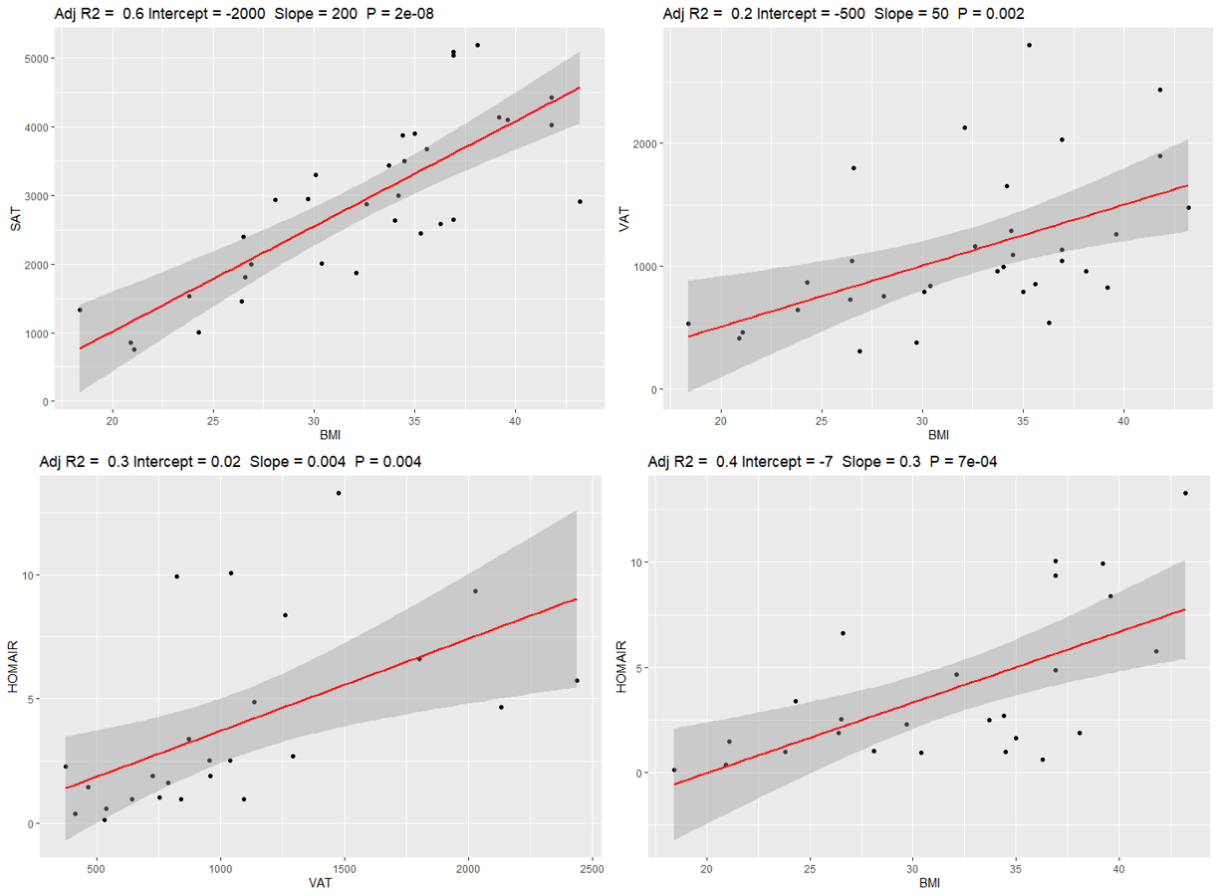
Scatter plots illustrate the significant associations between Homeostatic Model Assessment for Insulin Resistance (HOMA-IR), and body mass index (BMI), visceral and subcutaneous adipose tissue (VAT and SAT, respectively), based on linear regression models.

The Table 1 summarizes the clinical, demographics, and metabolic features of the sample of participants with available MRI, PiB, or AV-1451 scans. Based on the linear regression models in the overall sample, BMI was significantly associated with HOMA-IR (R2=0.37, p<0.001), VAT volume (R2=0.25, p=0.002), and SAT volume (R2=0.63, p<0.001). In addition, VAT volume was significantly associated with HOMA-IR (R2=0.27, p=0.004). The association between SAT volume with HOMA-IR trended towards but did not reach statistical significance (R2=0.10, p=0.07). However, VAT/SAT ratio was not significantly associated with BMI, or HOMA-IR (p=0.33, 0.50, respectively). The scatter plots for these relationships are shown in Figure 1.

There was a higher VAT/SAT ratio in males compared to females (p<0.001), while there was no difference between males and females in individual VAT or SAT volumes, BMI, or HOMA-IR. There was no difference in VAT, SAT, BMI, VAT/SAT ratio, or HOMA-IR by race.

### Relation of Alzheimer Disease Pathology with BMI, VAT/SAT ratio, and HOMA-IR

Partial Spearman’s correlation analyses showed statistically significant associations of VAT/SAT ratio with PiB SUVR in the right precuneus cortex (p=0.034, rho=0.55), controlling for sex. There were no significant associations between BMI and HOMA-IR with PiB SUVRs. Due to the difference in VAT/SAT ratio between males and females, associations of VAT/SAT ratio with right precuneus cortex PiB SUVR were separately assessed in males and females, showing only significance in males (rho=0.63, p=0.044), but not in females (rho=-0.48, p=0.166,).

There was no significant association between BMI or HOMA-IR and regional PiB SUVRs. Also, neither VAT/SAT ratio, BMI, nor HOMA-IR show significant associations with AV-1451 SUVRs.

**Figure 2.**
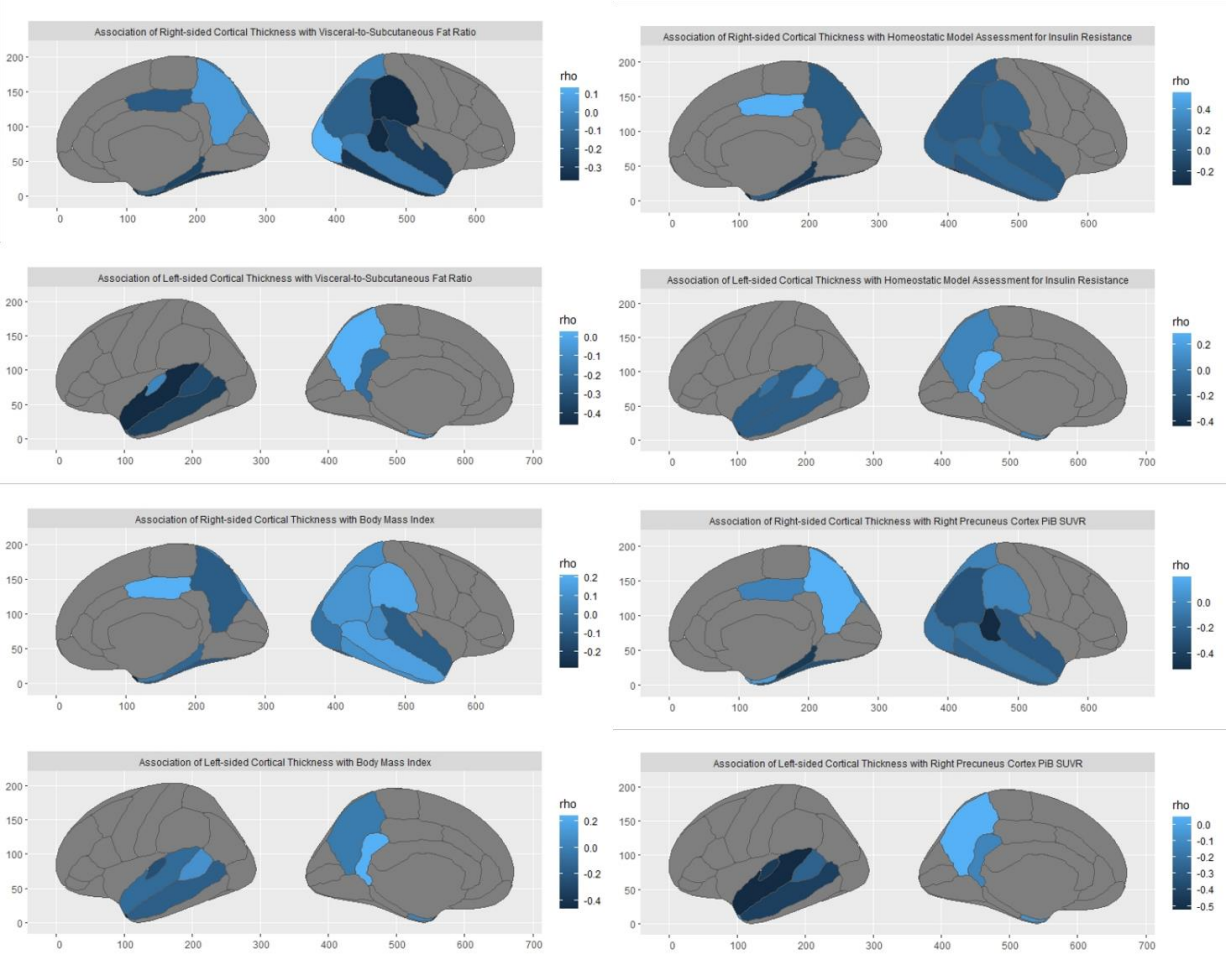
Illustration of brain regions with reduced cortical thickness in association with VAT/SAT ratio, BMI, HOMA-IR, and right precuneus PiB SUVR.

### Relation of Brain Volumes and Cortical Thicknesses with BMI, VAT/SAT ratio, and HOMA-IR

**Figure 3.**
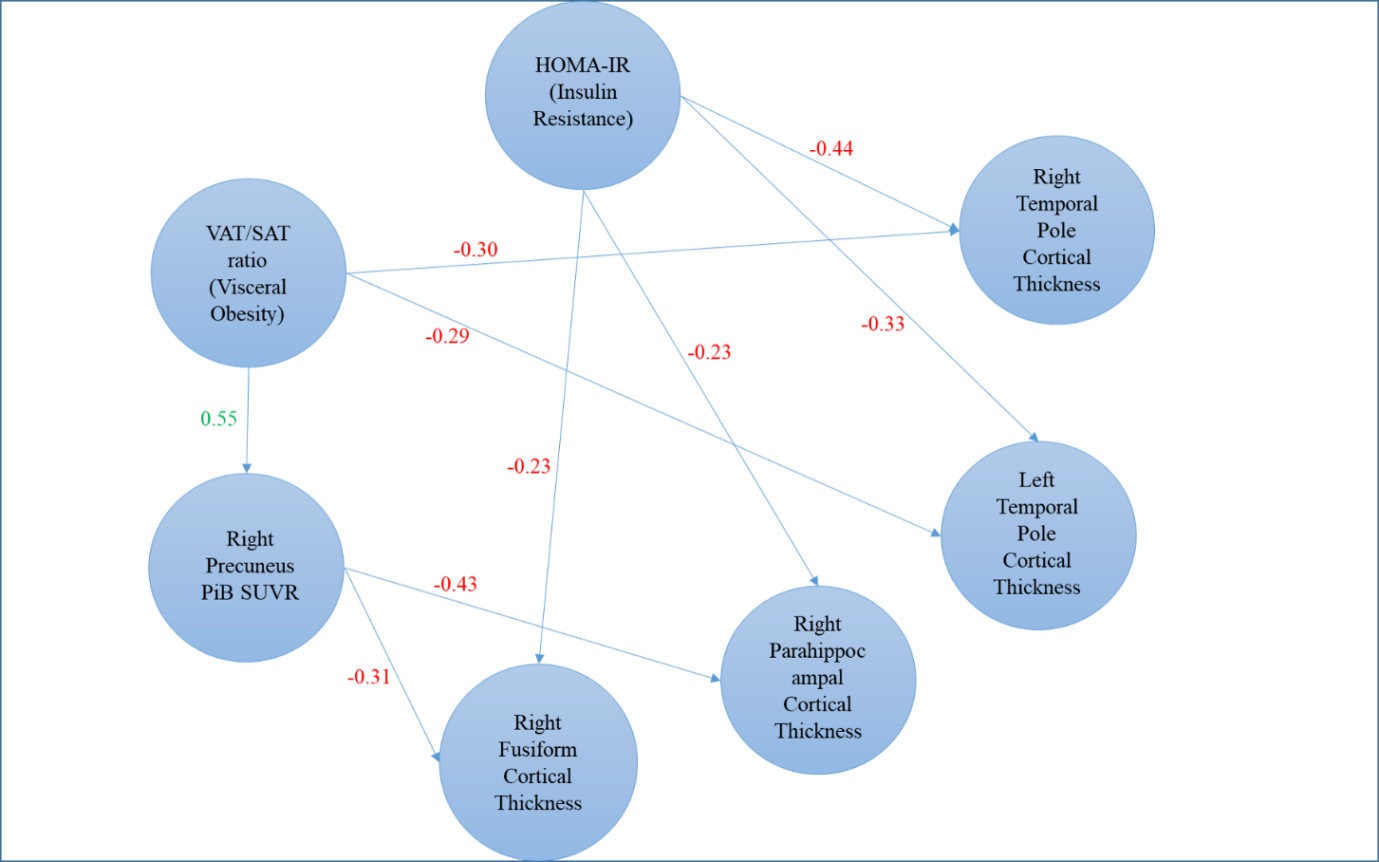
**Correlation model of the relationship among visceral-to-subcutaneous fat ratio, insulin resistance, right precuneus PiB SUVR, and right and left temporal pole, right fusiform, and right parahippocampal atrophy**. Path weights are partial correlation coefficients after adjusting for age and sex.

Spearman’s partial correlation analyses, with age and sex as covariates, and after correction for multiple comparisons, showed a statistically significant association between higher VAT/SAT ratio and cortical thickness in left superior temporal (p<0.00001), left and right banks of superior temporal sulcus (bankssts) (p<0.001), left middle temporal (p<0.001), left medial orbitofrontal (p<0.001), right fusiform (p<0.001), right inferior temporal (p<0.001), right supramarginal (p<0.001), right and left temporal pole (p=0.002), right parahippocampal (p=0.004), left isthmus cingulate (p=0.016), right posterior cingulate (p=0.029), right entorhinal (p=0.036), and right inferior parietal (p=0.038), as summarized in Table 2 and Figure 2-3.

In addition, higher BMI was associated with lower cortical thickness in the left temporal pole (p<0.001), right temporal pole (p=0.013), and left transverse temporal (p=0.025) cortices, after correction for multiple comparisons. Higher HOMA-IR was also associated with lower cortical thickness in the left and right temporal pole (p<0.001), right fusiform (p=0.017), and right parahippocampal (p=0.017) cortices after correction for multiple comparisons, as summarized in Table 2 and Figure 2, 3.

Based on Spearman’s partial correlation with intracranial volume, age, and sex as covariates, no statistically significant relationships were observed between BMI, VAT/SAT ratio, or HOMA-IR and brain volumes of interest.

### Relation of Cortical Thickness with Amyloid Pathology in the Right Precuneus

After correction for multiple comparisons, Spearman’s partial correlation analyses, with age and sex as covariates, revealed a statistically significant association between higher right precuneus PiB SUVR and cortical thickness in left middle temporal (p< 0.000001), right parahippocampal (p< 0.000001), left bankssts (p=0.002), left superior temporal (p<0.001), left medial orbitofrontal (p=0.010), right fusiform (p<0.001), right bankssts (p<0.001), right inferior temporal (p=0.008), right supramarginal (p<0.001), right inferior parietal (p=0.001), left transverse temporal (p=0.003), right parahippocampal (p=0.004), right superior temporal (p=0.003), left isthmus cingulate (p=0.038), right lateral occipital (p=0.038), and right middle temporal (p=0.035), as summarized in Table 2 and Figure 2, 3.

**Table 2:** Results of partial Spearman’s partial correlation analysis between cortical thickness in Alzheimer’s disease cortical signature areas and VAT/SAT ratio, BMI, and HOMA-IR, with sex and age as covariates.

	VAT/SAT ratio, rho (p-value)	BMI, rho (p-value)	HOMA-IR, rho (p-value)	Right Precuneus Cortex PiB SUVR, rho (p-value)
**Right inferior parietal**	**-0.155 (0.027)**	0.117 (1.0)	0.013 (1.0)	**-0.285 (0.003)**
**Right bankssts**	**-0.372 (0.00006)**	0.088 (1.0)	0.116 (1.0)	**-0.529 (0.00007)**
**Right precuneus**	0.055 (1.0)	**-0.087 (0.081)**	0.015 (1.0)	0.199 (1.0)
**Right superior parietal**	-0.028 (0.180)	0.096 (1.0)	0.019 (1.0)	**-0.034 (0.093)**
**Right lateral occipital**	0.133 (1.0)	-0.006 (0.229)	0.057 (1.0)	**-0.123 (0.023)**
**Left precuneus**	0.027 (1.0)	-0.020 (0.197)	0.063 (1.0)	0.046 (1.0)
**Left isthmus cingulate**	**-0.205 (0.010)**	0.243 (0.699)	0.285 (0.793)	**-0.127 (0.022)**
**Right entorhinal**	**-0.158 (0.025)**	0.023 (1.0)	0.052 (1.0)	0.100 (1.0)
**Right temporal pole**	**-0.296 (0.0009)**	**-0.286 (0.001)**	**-0.334 (0.00008)**	0.087 (1.0)
**Right middle temporal**	-0.057 (0.124)	0.153 (1.0)	0.079 (1.0)	**-0.135 (0.019)**
**Right parahippocampal**	**-0.261 (0.002)**	-0.044 (0.149)	**-0.234 (0.003)**	**-0.428 (0.000000008)**
**Right superior temporal**	**-0.282 (0.001)**	**-0.081 (0.089)**	-0.002 (0.159)	**-0.247 (0.001)**
**Right fusiform**	**-0.327 (0.0003)**	**-0.105 (0.063)**	**-0.229 (0.003)**	**-0.306 (0.001)**
**Right supramarginal**	**-0.362 (0.00009)**	0.176 (1.0)	0.051 (1.0)	-0.025 (0.103)
**Right inferior temporal**	**-0.323 (0.0004)**	0.102 (1.0)	-0.006 (0.153)	**-0.209 (0.004)**
**Right posterior cingulate**	**-0.172 (0.019)**	0.211 (0.851)	**0.560 (0.057)**	**-0.032 (0.094)**
**Left entorhinal**	-0.065 (0.112)	0.016 (1.0)	0.065 (1.0)	**-0.077 (0.051)**
**Left middle temporal**	**-0.375 (0.00005)**	**-0.171 (0.019)**	**-0.143 (0.020)**	**-0.421 (0.00000003)**
**Left superior temporal**	**-0.460 (0.0000005)**	**-0.107 (0.061)**	**-0.140 (0.021)**	**-0.522 (0.0009)**
**Left temporal pole**	**-0.294 (0.001)**	**-0.463 (0.0000003)**	**-0.437 (0.00000008)**	**-0.028 (0.099)**
**Left bankssts**	**-0.333 (0.0002)**	0.095 (1.0)	0.097 (1.0)	**-0.274 (0.0005)**
**Left medial orbital frontal**	**-0.390 (0.00002)**	-0.046 (0.145)	0.049 (1.0)	**-0.196 (0.005)**
**Left transverse temporal**	**-0.115 (0.054)**	**-0.249 (0.003)**	-0.026 (0.122)	**-0.445 (0.001)**

* The original p-values are reported, but only significant associations after correction for multiple comparisons are emboldened.

## DISCUSSION

This study, conducted in cognitively normal mid-life adults, demonstrates that increased abdominal VAT/SAT ratio, as a measure of visceral obesity, is associated with lower cortical thickness in multiple AD-signature areas, especially in temporal cortices which are mainly affected in late-onset Alzheimer disease. One other finding is that an increased abdominal VAT/SAT ratio is associated with higher amyloid uptake in the right precuneus cortex as measured by PiB SUVR in males but not in females. A similar pattern of association was observed between higher right precuneus cortex PiB SUVR and lower cortical thickness in AD signature areas. We also observed a significant association between higher HOMA-IR, as a measure of insulin resistance, and lower cortical thickness predominantly in temporal poles, and right parahippocampal and fusiform cortices but not amyloid pathology. Also, neither BMI, visceral obesity, nor insulin resistance were related to brain volumes or tau PET pathology in our sample.

In line with our findings showing lower cortical thickness in association with higher visceral compared to subcutaneous abdominal fat, prior studies have shown lower cortical thickness, in association with higher VAT [31, 32]. Specifically, Veit et al. observed lower cortical thickness in temporal cortex in the middle-aged population with higher VAT and BMI [32]. Also, other studies have shown smaller brain volumes, weaker brain connectivity, higher neuroinflammation, poorer memory performance, and increased risk of AD in association with increased VAT [33-36]. In addition to cortical atrophy, our data suggest a predilection to higher rates of amyloid pathology in the precuneus cortex of midlife males with higher visceral obesity. This association was observed in the precuneus cortex, which is affected by amyloid pathology early in the preclinical phase of AD [37], and along with the progression of AD pathology, a disruption in cholinergic activity ensues, leading to clinical AD [38]. A recent study revealed that participants with increased visceral fat metabolism, have higher amyloid uptake in multiple cortical regions, although the participants in this study had a diverse range of cognitive statuses and were older compared to our sample [17]. A prior study showed no significant association between BMI and amyloid cortical burden, based on Centiloid units, in the cognitively normal middle-aged population [10], which is in line with our non-significant findings on BMI association with amyloid pathology.

In this study, we used the VAT/SAT ratio, which in contrast to BMI or raw VAT volume determines the relative distribution of abdominal adipose tissue and is uniquely associated with cardiometabolic risk [20]. This is important especially because abdominal SAT is intrinsically distinct from VAT and even may be protective against metabolic derangements, including insulin resistance [39, 40]. In addition to visceral obesity, we showed the association of insulin resistance with neurodegeneration, but not molecular AD pathology. A large-scale retrospective cohort study on the midlife population showed a significantly higher risk for AD after a mean follow-up period of 7 years in patients with the highest quartiles of triglyceride glucose index, as a marker of insulin resistance [41]. Also, higher HOMA-IR in midlife was associated with lower cerebral glucose uptake in several cortical regions especially in the left middle temporal lobe, an AD-signature area, and predicted worse cognitive performance [42]. Although brain volume changes in areas such as the hippocampus and middle temporal lobe are observed in association with diabetes mellitus and insulin resistance in midlife [43, 44], in a sample of middle-aged to older non-demented adults, no difference was observed in amyloid PET SUVRs or amyloid PET-positivity between diabetic and non-diabetic groups, while only among the amyloid PET-positive participants, tau PET SUVRs were higher in the diabetic group compared to non-diabetic group [45].

Our results may indicate early neurodegenerative processes in AD-related cortical regions transpiring in the midlife population with higher visceral adiposity and insulin resistance, even without any overt cognitive impairment. While our study does not directly reveal specific underlying mechanisms of our observations, visceral adiposity may contribute to the progression of neurodegeneration through increased insulin resistance. Postulated mechanisms include lowering levels of brain-derived neurotrophic factor (BDNF), which is involved in neurodegeneration through altered neural plasticity [46]. Another suggested possible pathway is increased production of pro-inflammatory cytokines, oxidative stress, and advanced glycation end-products accumulation, increased apoptosis of neuronal cells, and cerebral microvascular disease, starting as early as midlife [47, 48].

Although we did not observe any significant association between amyloid or tau deposition and insulin resistance, impaired insulin signaling can also lead to a reduction in the clearance of amyloid plaques and promotion of tau hyperphosphorylation [48]. In addition to insulin resistance, visceral obesity through a combination of other unknown mechanisms may have contributed to the increased amyloid uptake and neurodegeneration observed in our results. One hypothesis is that an imbalance in the distribution of abdominal adipose tissue could lead to dysregulation of related secretions, such as leptin, and inflammatory cytokines, that could contribute to the development of Alzheimer disease pathology and neurodegeneration by inducing oxidative stress, and inflammation and potentially other unknown mechanisms [49, 50]. Peripheral inflammation signals directly transmitted to the brain via nerve afferents, may trigger alterations in amyloid transport, increasing aggregation [51]. Further activation of microglia by amyloid is thought to lead to impairing their clearance functions [51]. Leptin resistance could also directly contribute to beta-amyloid production and tau phosphorylation, due to a large number of leptin receptors in the hippocampus, that can propagate amyloid pathology through connections to the precuneus [49, 52, 53].

It is worthy of note that these observations were made in our middle-aged sample. In the elderly, however, lower BMI and frailty may be related to preclinical changes in weight and altered nutritional intake due to cognitive dysfunction, and the comorbidities associated with Alzheimer disease [54, 55], making the pattern of associations distinct from midlife.

Our sex difference findings also reflect increasing interest in this area, as sex-related differences in body fat content associations with the risk of AD have been studied in late life. For instance, a diffusion tensor imaging study in older adults showed more prominent associations between BMI and white matter diffusion metrics in males [56]. In line with our findings, estradiol levels are shown to alleviate the negative association between VAT and brain structural network covariance in middle-aged females, but not for males [36], leaving males with a higher susceptibility due to increased VAT. Interestingly, it was shown by an animal study that SAT lipectomy eliminated resistance to proinflammatory cytokines in the microglia of female mice irrespective of sex hormone levels, highlighting the role of fat distribution in determining sex differences in susceptibility to obesity-induced neuroinflammation [57]. Additionally, the rate of lipolysis and free fatty acid mobilization from visceral fat to the systemic venous system is higher in males compared to females, which is due to the differential fat cell volume and the response to adrenoreceptors in males and females [58].

In line with longitudinal studies in preclinical AD and autosomal dominant AD, showing that amyloid pathology is present years before tau pathology and symptoms onset [59, 60], our study in the cognitively normal midlife population showed no association of obesity, abdominal fat metrics, or insulin resistance with brain tau uptake. Prior evidence based on the amyloid cascade hypothesis shows that beta-amyloid plaques enhance endogenous tau accumulation which further promotes tau neurofibrillary tangles accumulation [61]. Thus, the association of visceral obesity with beta-amyloid pathology in midlife, most probably precedes tau accumulation and cognitive decline. In later stages, however, when amyloid burden reaches a significant level, obesity and metabolic derangements propagate tau tangles deposition, based on prior studies [45].

Our findings, along with prior evidence, point to the importance of lifestyle modifications to prevent the incidence of clinical AD. For instance, it has been shown that physical exercise can alleviate amyloid aggregation through mechanisms like a reduced expression of beta-secretase, as well as promoting clearance of beta-amyloid aggregates through activation of neprilysin and insulin-degrading enzyme [62]. Besides physical activity, the Mediterranean diet, social engagements, and other lifestyle modifications can improve cognitive reserve through amyloid-independent pathways and prevent or delay clinical dementia [63].

This study has multiple strengths. We recruited a diverse midlife sample consisting of participants from different racial groups, with a wide range of BMI (18.4-43.2 kg/m2). Employing abdominal MRI for measurement of VAT and SAT in conjunction with BMI and insulin resistance measures, enabled us to capture body composition, adipose tissue distribution, and the associated metabolic alterations more accurately. We used the visceral-to-subcutaneous fat ratio, which is used as a valid marker of visceral obesity and is associated with cardiovascular risk, to account for the adipose tissue distribution and the potentially beneficial effects of subcutaneous fat. In addition, we deployed both amyloid and tau PET scans as well as MRI data for simultaneous assessment of AD-related pathology and neurodegeneration. To further enhance our understanding, we are looking into the brain microstructural alterations and neuroinflammation based on diffusion spectrum imaging, besides levels of inflammatory markers in circulation, in association with midlife obesity.

However, our study has several limitations. This is a pilot study, and the sample size may not be large enough to exclude random errors or to provide enough power to capture the present differences. This study is ultimately projected to recruit a total of 120 participants, enabling us to eventually overcome this limitation. Another limitation is the cross-sectional design of this study, which does not allow for examining the causality of changes.

Overall, this study highlights the association of abdominal visceral adiposity with beta-amyloid, but not tau, pathology in the precuneus cortex, an area affected early during the progression of AD pathology, in middle-aged males. Also, midlife participants with higher BMI, visceral adiposity, and insulin resistance tended to have lower cortical thickness in AD cortical signature areas especially temporal regions, which are involved with cognitive function. This highlights the importance of preventing obesity, especially abdominal adiposity, through lifestyle modifications, early in midlife. However, further longitudinal studies are warranted to examine the role of metabolic and lifestyle-related factors in the propagation of AD pathology.

## Supplementary Materials

The Supplementary data can be found online at: www.aginganddisease.org/EN/10.14336/AD.2023.0707.


