# Supplementary material for: Alzheimer Disease Pathology and Neurodegeneration in Midlife Obesity: A Pilot Study
Source: Aging Dis. 2024 Aug 1;15(4):1843–54. doi: 10.14336/AD.2023.0707 (PMC11272197; doi:10.14336/AD.2023.0707)
Supplement: Supplementary file 1 [file AD-15-4-1843-s.pdf]

SUPPLEMENTARY DATA

Alzheimer Disease Pathology and Neurodegeneration in Midlife Obesity: A Pilot Study

Mahsa Dolatshahi, Paul K. Commean, Farzaneh Rahmani, Jingxia Liu, LaKisha Lloyd, Caitlyn Nguyen, Nancy Hantler, Maria Ly, Gary Yu, Joseph E. Ippolito, Claude Sirlin, John C. Morris, Tammie L.S. Benzinger, Cyrus A. Raji

© 2023. Dolatshahi M et al. Published online at http://www.aginganddisease.org/EN/10.14336/AD.2023.0707

SUPPLEMENTARY DATA

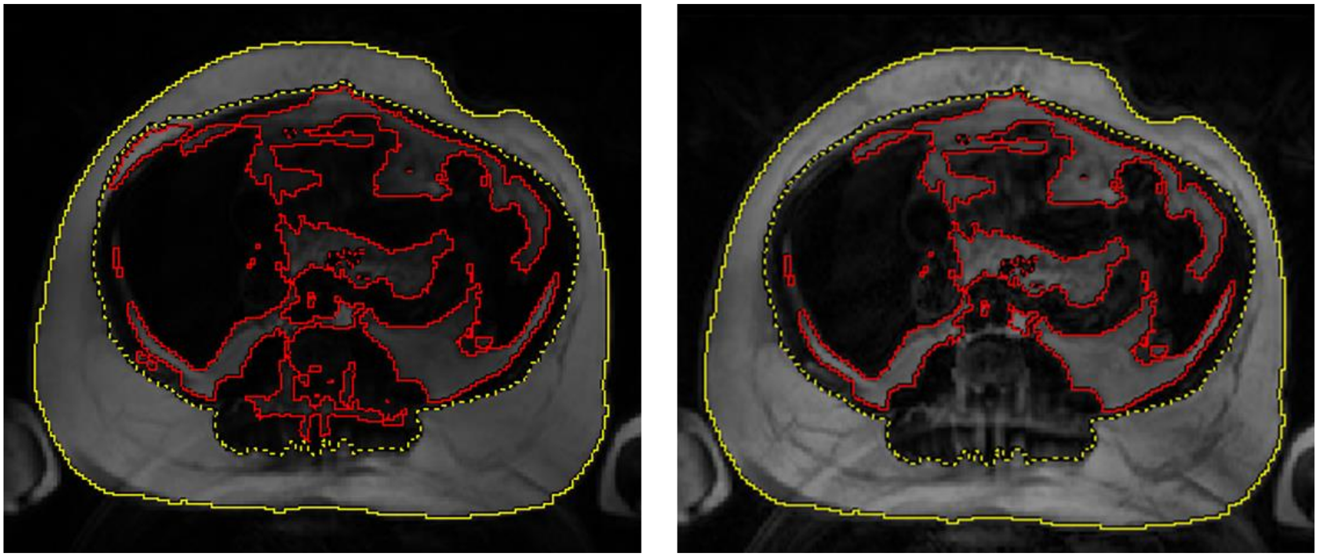

Supplementary Figure 1. After automatic segmentation of subcutaneous and visceral fat by Voxa (left), the segmentations were inspected and manually edited, including manual removal of fat in the spinal region (right).

Supplementary Table 1. Cortical signature FreeSurfer regions in autosomal dominant and late onset AD.

ADAD Cortical Signature, Right Hemisphere	ADAD Cortical Signature, Left Hemisphere	LOAD Cortical Signature, Right Hemisphere	LOAD Cortical Signature, Left Hemisphere
**inferior parietal**	precuneus	entorhinal	entorhinal
**bankssts**	isthmus cingulate	temporal pole	middle temporal
**precuneus**		middle temporal	superior temporal
**superior parietal**		parahippocampal	temporal pole
**lateral occipital**		precuneus	bankssts
		superior temporal	medial orbital frontal
		fusiform	transverse temporal
		inferior parietal	precuneus
		bankssts	
		supramarginal	
		inferior temporal	
		superior parietal	
		posterior cingulate	

© 2023. Dolatshahi M et al. Published online at http://www.aginganddisease.org/EN/10.14336/AD.2023.0707
